# Supplementary material for: Intention to receive a COVID-19 vaccine: results from a population-based survey in Canada
Source: BMC Public Health. 2021 May 29;21:1017. doi: 10.1186/s12889-021-11098-9 (PMC8164402; doi:10.1186/s12889-021-11098-9)
Supplement: Supplementary file 1 — Additional file 1. Survey Questions. Contains a list of all survey questions asked to respondents, which are relevant to the analysis described in this article. [file 12889_2021_11098_MOESM1_ESM.docx]

Additional File 1:

Survey Questions

Core Module

1. Are you 25-69 years old (inclusive)?
   1. Yes
   2. No (End Survey)
2. Are you a resident of BC?
   1. Yes
   2. No (End survey)

The next series of questions ask about presence or absence of symptoms for COVID-19. Symptoms of COVID-19 may include

1. Fever >= 38
2. Feverishness
3. Chills
4. Cough
5. Fatigue
6. Muscle pain
7. Joint pain
8. Sore throat
9. Nasal congestion
10. Shortness of breath
11. Wheezing
12. Other respiratory symptom
13. Headache
14. Alteration to taste
15. Alteration to smell
16. Nausea/vomiting
17. Abdominal pain
18. Diarrhea.
19. Do you think you have had COVID-19?

- No
- Yes

1. *If yes to question #3,* Why do you think you have had COVID-19? Please check all that apply

- Symptoms (cough, fever, shortness of breath, etc.)
- Nasal/throat test result
- Health care provider
- Contact with case

1. *If yes to question #3,* Where do you believe you came into contact with a case of COVID-19? If you are unsure where, please choose the most likely exposure environment.

- Household
- High risk environment (including healthcare setting)
- Occupational setting
- Other local setting – Specify:
- Travel
- Don’t know

1. *If yes to question #3,* Did you have a nasal/throat swab to test for COVID-19?

- Yes
- No

1. *If yes to question #6,* What month did you have the COVID-19 test?
   1. January
   2. February
   3. March
   4. April
   5. May
   6. June
   7. July
   8. August
   9. September
   10. October

November

1. *If yes to question #6,* Was it early, mid, or late [**month from above**]?

- Early
- Mid
- Late

1. *If yes to question #6,* What was the result of your COVID-test?

- Positive
- Negative
- I have not received the results yet

1. *If “positive” to question #9,* Did you require hospitalization due to COVID-19?

- No
- Yes

1. *If yes to question #10,* What was your admission date? *If you are not sure, leave blank*
2. *If yes to question #10,* What was your discharge date? *If you are not sure, leave blank*
3. *If yes to question #10*, Did you require ventilation because of COVID-19?

- Yes
- No

1. *If yes to question #3,* What were your symptoms while you were sick/thought you were sick? *Select all that apply*

- Fever
- Cough
- Fatigue
- Muscle pain
- Sore throat
- Shortness of breath
- Headache
- Decreased sense of smell
- Nausea/vomiting
- Diarrhea
- Other symptoms

1. *If endorses any in question #14,* Approximately when did the symptoms start?

- January
- February
- March
- April
- May
- June
- July
- August
- September
- October
- November

1. *If endorses any in question #14,* Approximately when during [**pipe in from above**] did the symptoms begin?

- Early
- Mid
- Late

1. *If yes to question #3,* Did you require any over the counter medication for any symptoms (cough, fatigue, fever, pain)?

- Yes
- No

1. *If endorsed any in question #14,* Are your symptoms resolved?

- Yes
- No

1. *If yes to question #3,* Are you currently in isolation?

- Yes
- No

1. *If yes to question #3,* Where did you stay during the time you were sick/thought you were sick? *Check all that apply*

- I did not change where I was staying
- In home isolation (separated from other household members)
- In home isolation (not separated)
- In a rental or hotel
- In a hospital
- Other

1. *If “contact with case” in question #4*, Where did your contact with a known COVID-19 positive person occur?

- Household
- High risk environment (including a health care setting)
- Occupational setting
- Other local setting
- Travel

1. *If “high risk environment” in question #21*, In what high risk environment do you believe you came in contact with a COVID-19 positive person?

- Hospital
- Nursing home
- Public health clinic
- Shelter
- COVID testing facility
- Other – specify:

1. *If “occupational setting” in question #21*, What occupational risk may have caused you to be in contact with a COVID-19 positive individual?

- health care worker - direct patient contact,
- health care worker - indirect patient contact,
- law enforcement or first responder,
- vulnerable population service provider,
- food and agricultural service provider (grocery, pharmacy, liquor,

other household products),

- transportation worker (bus driver, sky train operators, west coast express, seabus, handi-dart, customer facing airport employees, etc)
- Childcare worker
- Correctional officer
- Teacher/other school staff
- Hairdresser/Barber
- Aesthetician
- Flight attendant
- Factory worker

1. Have you traveled outside of British Columbia since January 2020?

- Yes
- No

1. *If yes to question #24,* Where and how many times you have traveled since January 2020? Check all that apply.

- I traveled outside of BC (within Canada) once
- I traveled outside of BC (within Canada) multiple times
- I traveled outside of Canada once
- I traveled outside of Canada multiple times

1. What is your current age (years)?
2. What sex were you assigned at birth?

- Female
- Male
- Intersex
- Prefer to self-describe – Specify:
- Prefer not to answer

1. Which best describes your current gender identity?

- Man
- Woman
- Two-Spirit
- Non-Binary, GenderQueer, Agender, or similar identity
- Prefer to self-describe – Specify:
- Prefer not to answer

1. *If question #27 and question #28 are not [“male” and “man”] or [“female” and “woman”],* What gender do you currently live as in your day-to-day life?

- Man
- Woman
- Sometimes man, sometimes woman
- Something other than man or woman

1. What is your sexual orientation?

- Asexual
- Bisexual
- Demisexual
- Gay/Lesbian
- Heterosexual
- Pansexual
- Prefer to self-describe – specify: ______
- Prefer not to answer

1. Are you an Indigenous person originating from North America? *Check any that apply.*

- First Nation
- Métis
- Inuit
- Non-status First Nations
- Other Indigenous
- Prefer not to answer
- I am not an Indigenous person originating from North America

1. *If endorse any Indigenous group in question #31,* Do you live on or off reserve?

- On-reserve
- Off-reserve
- Prefer not to answer

1. Do you consider yourself to be? *Please check all that apply to you.*

- Black African (e.g., Nigerian, Somali)
- Black Caribbean (e.g., Haitian)
- Black Other (e.g., Black Canadian)
- White
- Chinese or Taiwanese
- Filipino
- Japanese
- Korean
- Latin American (e.g., Chilean, Costa Rican, Mexican)
- South Asian (e.g., Indian, Bangladeshi, Pakistani, Punjabi, and Sri Lankan)
- Southeast Asian (e.g.,Cambodian, Laotian, Malaysian, Vietnamese)
- Arab (e.g., Egyptian, Kuwaiti, and Libyan)
- West Asian (e.g. Iraqi, Isreali, Lebanese, Afghani, Iranian)
- Central Asian (e.g., Kazakhstani, Krgyzstani, Tajikistani, Turkmenistani)
- Multiple races / Multiracial / “Mixed”
- Prefer not to answer
- Other, please specify: _____________________________

1. What is your current legal status in Canada?

- Canadian citizen – born in Canada
- Naturalized Canadian citizen
- Landed immigrant/Permanent Resident
- Refugee/Protect Person/Refugee claimant
- Here with Temporary Work Papers
- Here with Humanitarian and Compassionate approval
- Here as a visitor
- Here on a Student Visa
- Undocumented/Illegal Immigrant
- Other – Please specify:
- Don't know
- Prefer not to answer

1. *If “landed immigrant” in question #34,* in what year did you first become a landed immigrant to Canada? Please enter the year as 4 digits (YYYY).

- YYYY

1. What is your marital status?

- Never legally married – currently single
- Never legally married – living with partner < 12 months
- Never legally married – in a relationship
- Legally married
- Separated, but still legally married
- Common law
- Divorced
- Widowed

1. How many adults (18+ years) live in your household?
2. How many children under the age of five live in your household?
3. How many children age five to seventeen live in your household?
4. What is your highest achieved education level?
5. No certificate, diploma or degree
6. Secondary (high) school diploma or equivalency certificate
7. Apprenticeship or trades certificate or diploma
8. College, CEGEP or other non-university certificate or diploma
9. University certificate or diploma below bachelor level
10. University certificate, diploma or degree at bachelor level or above
11. Are you currently attending school, college, university or other?
12. Yes
13. No
14. Do you expect to be a student this fall?
15. Yes
16. No
17. *If “yes” to question #42,* Do you expect to be a full-time or part-time student in the fall?
18. Full-time
19. Part-time
20. Don’t know
21. Do you consider yourself an essential worker? *Will direct to either question #45 or #46*

- No
- Yes, health worker
- Yes, other essential worker

1. *If “health worker” in question #44,* Type of health worker:

- Physician
- Nurse
- Occupational Therapist
- Physical Therapist
- Respiratory Therapist
- Care Aid/Personal Care Worker
- Dietician
- Healthcare worker - indirect patient contact (stocking shelves, cleaners, cafeteria, mechanics/engineers, etc)
- other – specify:

1. *If “Yes, other essential worker” in question #44,* Type of other essential worker:

- Law enforcement or first-responder
- Vulnerable population service provider (e.g. social worker, youth worker)
- Food and agriculture service provider (grocery, pharmacy, liquor, other household products)
- Transportation worker (bus driver, sky train operations, west coast express, seabus, handi-dart, customer facing airport employees, etc)
- Engineer or construction worker
- other – specify

1. The section asks about whether you've ever been diagnosed with a chronic health condition. We are interested in long-term conditions which are expected to last or have already lasted 6 months or more and that have been diagnosed by a health professional. Please check all that apply.

   Has a doctor ever told you that you have...

|  | Yes | No | Prefer not to answer |
| --- | --- | --- | --- |
| Asthma (A common lung disorder in which inflammation causes the bronchi to swell and narrow the airways, creating breathing difficulties) |  |  |  |
| COPD or emphysema (Chronic Obstructive Pulmonary Disease or COPD a long-term, progressive disease of the lungs that primarily causes shortness of breath due to over-inflation of the alveoli (air sacs in the lung)) |  |  |  |
| Chronic lung disease (A disorder that can be caused by smoking tobacco or inhaling air pollutants that usually develops slowly, may get worse over time, and affects the lungs and other parts of the respiratory system) |  |  |  |
| Insulin resistance/pre-diabetes/borderline diabetes (A condition in which a decreased response to insulin by a person's body tissues results in the production of larger quantities of insulin to maintain normal levels of glucose in the blood) |  |  |  |
| Diabetes (A disease in which there is reduced production of, or response to, insulin so that glucose cannot be absorbed into the cells of the body) |  |  |  |
| High blood pressure/hypertension (Abnormally high arterial blood pressure that is usually indicated by an adult systolic blood pressure of 140 mm Hg or greater or a diastolic blood pressure of 90 mm Hg or greater) |  |  |  |
| Heart disease (A disease in which an accumulation of plaque in the vessels that supply blood to the heart causes impaired heart functioning) |  |  |  |
| Coronary Artery Disease/Myocardial Infarction/Heart Attack (Deprivation of oxygen, usually due to blockage of a diseased coronary artery, damages the heart and causes chest pain) |  |  |  |
| Heart Failure (A condition in which the heart fails to pump adequate amounts of blood to body tissues, resulting in an accumulation of blood returning to the heart from the veins) |  |  |  |
| Cardiac arrhythmia/Atrial Fibrillation (A condition in which the normal rhythmical contractions of the heart are replaced by rapid irregular twitchings of the muscular wall) |  |  |  |
| Stroke (Sudden impairment or loss of consciousness, sensation, and voluntary motion that is caused by rupture or obstruction of a blood vessel supplying the brain and is accompanied by permanent damage of brain tissue) |  |  |  |
| Deep vein thrombosis/Pulmonary Embolism (A condition marked by the formation of a clot within a deep vein that may be accompanied by symptoms and that is potentially life threatening if the clot dislodges and obstructs the pulmonary vasculature) |  |  |  |
| Peripheral Vascular Disease (A disease affecting blood vessels outside of the heart and especially those vessels supplying the extremities) |  |  |  |
| High cholesterol (Too much cholesterol in the blood, usually considered above 6.2 mmol/L) |  |  |  |
| Liver disease (i.e., fatty liver) (An abnormal condition of the liver that is marked by excess fat accumulation in the liver cells and that is typically associated with obesity, malnutrition, rapid weight loss, excessive alcohol consumption, high blood fat content, or diabetes) |  |  |  |
| Liver cirrhosis (Compromised liver structure and function that results from liver cell death, inflammation, and scar tissue) |  |  |  |
| Renal problem/Kidney problem. (Impaired ability of the kidneys to perform vital functions that can be due to an acute event or a chronic condition or disease) |  |  |  |
| Autoimmune Disorder (A disorder caused by a reaction of the immune system against the organs or tissues of the body) |  |  |  |
| Pneumonia that was confirmed using chest x-rays (An acute disease that is marked by inflammation of the lung tissue and typically causes fever, chills, cough, difficulty breathing, fatigue, chest pain, and reduced lung expansion) |  |  |  |
| Chronic neurological or neuromuscular disorder (A long-lasting disorder that is caused by a dysfunction in part of the brain or nervous system and that results in physical or psychological symptoms or that affects voluntary muscle control) |  |  |  |

1. Are you a person living with a disability?
2. Yes
3. No
4. Prefer not to answer
5. Are you a person living with HIV?
6. Yes
7. No
8. Don’t know
9. Prefer not to answer
10. Has a doctor ever told you that you have cancer (excluding skin malignancies)?
11. Yes
12. No
13. Prefer not to answer
14. *If yes to question #50*, What type(s) of cancer were you diagnosed with? Please select all that apply.
15. Anal
16. High Grade Anal precancer (Anal Intraepithelial Neoplasia, AIN 2 or 3)
17. Bladder
18. Bone
19. Breast
20. Cervical (“HPV”)
21. High Grade Cervical precancer * (Cervical Intraepithelial Neoplasia or CIN 2 OR 3)
22. Colon or Rectum
23. Endometrial (ie. of the uterus)
24. Kaposi Sarcoma
25. Kidney
26. Liver
27. Lung
28. Lymphoma / leukemia
29. Oral or pharynx
30. Ovarian
31. Skin (melanoma, basal, squamous cells)
32. Stomach or Small Bowel
33. Thyroid
34. Vulvar
35. High Grade Vulvar or vaginal precancer (Vulvar or Vaginal Intaepithelial Neoplasia, VIN or VaIN 2 or 3)
36. Other, please specify:
37. Don't know
38. Prefer not to answer
39. During the COVID-19 pandemic (mid March to now) were you due for, or in need of, any screening appointments? *Select all that apply.*
40. Pap test
41. Colonoscopy
42. Mammogram
43. Other, please specify: ______
44. *If any checked off in question #52,* Did you attend the above appointments for which you were due?
45. I attended all
46. I attended some
47. I attended none
48. Prefer not to answer
49. *If some/none,* Why did you not attend the appointments?
50. My doctor or clinic was not accepting in-person appointments
51. My appointment was considered non-urgent
52. Worried about visiting doctor or doctors’ office
53. Other, please specify: _____________
54. Are you pregnant?

- Yes
- No

1. *If yes to above*, How many weeks pregnant are you? *If you don’t know, please leave blank.*
2. *If no to question #55,* Have you been pregnant at anytime since March 2020?

- Yes
- No

1. *If yes to question #57,* Are you 6 weeks or less postpartum?

- Yes
- No

1. *If yes to question #57,* Since March 2020 have you suffered from any miscarriages?
2. Yes
3. No
4. Please enter your height in feet and inches
5. Please enter your weight in pounds

We would now like to ask you some questions about your feelings surrounding vaccines, and whether or not your thoughts about vaccines for your health have changed since the COVID-19 Pandemic

1. If a COVID-19 vaccine were to become available to the public, and recommended for you, how likely are you to receive it?

- Very unlikely
- Unlikely
- Neutral
- Somewhat likely
- Very likely

1. Do you receive all recommended vaccines when offered to you by health care professionals?

- Yes
- No
- Don’t know

1. Thinking back to before the beginning of the pandemic (December 2019), how much did you value vaccines?

- No value
- Very little value
- Neutral
- A little value
- Valued a lot

1. Today, how much do you value vaccines?

- No value
- Very little value
- Neutral
- A little value
- Valued a lot

1. Have you received the influenza vaccine in the past 5 years?

- Never
- 1-2 times
- 3-4 times
- Every year
- Don’t know

1. Do you intend to receive the influenza vaccine in the fall of 2020?

- Yes
- No
- Don’t know
- I have already received the vaccine

1. *WHO Vaccine Hesitancy Scale (Vaccine Confidence Scale)* [Section Instructions:] *This section will ask you about your opinion about vaccines, in general.* *Please indicate how much you agree or disagree with each statement below.*

|  | Strongly disagree | Somewhat disagree | Neutral | Somewhat agree | Strongly agree |
| --- | --- | --- | --- | --- | --- |
| Childhood vaccines are important for a child’s health |  |  |  |  |  |
| Getting vaccines is a good way to protect children from disease |  |  |  |  |  |
| Childhood vaccines are effective |  |  |  |  |  |
| Having a child vaccinated is important for the health of others in my community |  |  |  |  |  |
| All childhood vaccines offered by the BC immunization program in my community are beneficial |  |  |  |  |  |
| The information I receive about vaccines from the vaccination program is reliable and trustworthy |  |  |  |  |  |
| Generally, I do what my doctor or health care provider recommends about vaccines |  |  |  |  |  |
| New vaccines carry more risks than older vaccines |  |  |  |  |  |
| I am concerned about potential serious adverse effects of vaccines |  |  |  |  |  |
| Children do not need vaccines for diseases that are not common anymore |  |  |  |  |  |
| The risk associated with diseases is larger than the risk associated with vaccinations |  |  |  |  |  |

Vaccine Module

*Assuming that a safe and effective COVID-19 vaccine was available and recommended, please indicate how much you agree or disagree with the following statements:*

Attitudes

[*5-point scale: 1 strongly disagree, 3 neutral, 5 strongly agree]*

1. A COVID-19 vaccine would be beneficial
2. A COVID-19 vaccine would be beneficial for children
3. A COVID-19 vaccine would be beneficial for individuals 60-years and older
4. COVID-19 is a serious illness
5. A COVID-19 vaccine would be beneficial for the health of my community
6. A COVID-19 vaccine would be safe
7. A COVID-19 vaccine would be effective in preventing COVID-19
8. A COVID-19 vaccine should be mandatory

*Please answer the following questions, with the assumption that a safe and effective COVID-19 vaccine is available and recommended.*

Social Norms

Q77-80, 82, 84, 86, 88, 90, 92, 94: [*5-point scale: 1 strongly disagree, 3 neutral, 5 strongly agree]*

Q81, 83, 85, 87, 89, 91, 93: [*5-point scale: 1 strongly disapprove, 3 neutral, 5 strongly approve]*

1. Most people who are important to me would think that I should receive the COVID-19 vaccine
2. People who are important to me would expect me to receive the COVID-19 vaccine
3. I would feel under social pressure to receive a COVID-19 vaccine
4. Everyone I know would get the COVID-19 vaccine
5. My family physician (or other primary Health Care Provider) would approve/disapprove of me receiving a COVID-19 vaccine
6. What my family physician (or other primary Health Care Provider) thinks is important to me
7. The BC Public Health Officer would approve/disapprove of me receiving the COVID-19 vaccine
8. What the BC Public Health Officer recommends is important to follow
9. My coworkers would approve/disapprove of me receiving the COVID-19 vaccine
10. What my coworkers think is important to me
11. My employer/work institution would approve/disapprove of me receiving the COVID-19 vaccine
12. What my employer/work institution thinks is important to me
13. The educational institution (elementary/high school/college/university) that I or my children attend/are associated with would approve/disapprove of me receiving the COVID-19 vaccine
14. What my school/children’s school thinks is important to me
15. My friends would approve/disapprove of me receiving the COVID-19 vaccine
16. What my friends think is important to me
17. My family would approve/disapprove of me receiving the COVID-19 vaccine
18. What my family thinks is important to me

Behavioural Control

Q95-98: [*5-point scale: 1 strongly disagree, 3 neutral, 5 strongly agree]*

Q99: [*Sliding scale: 1 numerous, 5 some, 10 very few]*

For the next 3 questions: If a COVID-19 vaccine was offered and publicly funded and available, like the flu shot …

1. … It would be difficult to receive the COVID-19 vaccine
2. … I could easily receive a COVID-19 vaccine if I wanted to
3. … It would be completely up to me whether I received the COVID-19 vaccine
4. How much control do you feel you would have over whether you receive a COVID-19 vaccine?

- Very little control
- Not much control
- Neutral
- Some control
- A lot of control

1.
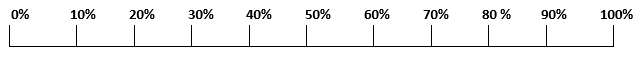
The number of events outside my control which would prevent me from having a COVID-19 vaccination are:

**0 5 10**
